# On Catalepsy

**Published:** 1830-07-01

**Authors:** 


### XXXVI.

### CATALEPSY.

A case of this strange disease having recently appeared in the Royal Infirmary of Edinburgh, the clinical remarks of Dr. Duncan have been collected and published in a weekly contemporary. We shall take a short notice of this case, the more especially as one not very dissimilar is at the present moment under our care.

The northern patient was a young woman 25 years of age, who became affected with catalepsy about the first of February of the present year, and was received into the infirmary on the 5th of the same month. She was first discovered sitting in the kitchen like a statue, stiff and insensible—yet her limbs yielding easily to any force which was applied, and remaining in any position in which they were put. When received into hospital, there was complete loss of voluntary motion occurring in paroxysms, and continuing about ten minutes at a time, during which the limbs retained a certain degree of flexibility, but resisted, to a certain extent, the application of external force. During the fits there were slight convulsive writhings of the muscles—the eyes were not turned up, as in epilepsy, but rolled about, the pupils dilating and contracting. Before the commencement of the paroxysms she feels a fluttering about the heart, general faintness, and heaviness about the head. After the paroxysm is over, she feels a universal soreness of the limbs, as epileptics do, attended with a severe pain in a certain part of the spine, which is tender to the touch. The tongue was white, pulse 70 in the intervals; but rapid and feeble during the fits. *The catamenia had appeared at the proper period, before the commencement of the disease, but were suddenly suppressed by cold.* Some inhuman sceptic had torn two pieces of skin off her hands in one of these paroxysms!

The case continued much the same, not at all relieved, till the 15th of February, when severe pain was complained of in the right iliac region, which though reasonably considered as nervous by Dr. Duncan, was treated by leeches, lest inflammation should actually exist. During the cataleptic paroxysms she was, of course, free from pain; but the moment they were over, she screamed out from the violence of the agony. The iliac region was extremely tender to the touch; but no tumour was perceptible. The leeches bled freely—a fœtid enema was thrown up, but speedily returned. Powerful anodynes were given both by the mouth and anus, yet without any effect. A warm bath and a still more overwhelming opiate allayed the agony. In the course of little more than 24 hours she had taken 125 drops of Battley's sedative liquor, 120 drops of common laudanum, two grains of solid opium, besides castor, æther, valerian, and most of the stinking drugs in the pharmacopœia. These and other circumstances induced Dr. Duncan to conclude that the disease was of a purely nervous character, and, in fact, that it was one of those singular and strange forms which hysteria occasionally assumes.

After this the complaint took on another new but temporary form. On the 3d of March the patient received some unpleasant intelligence, and requested her dismissal. This being refused, a very severe paroxysm occurred, and lasted three quarters of an hour. Several of these attacks came on in the course of a few hours.

"During the last fit, the mental functions appeared to be maintained in considerable precision; she sung psalms, and prayed with exactness and correct modulation; and once having stopped short in repeating the Lord's prayer, she, in a few seconds, resumed it where she left off. The most extraordinary circumstance, however, was the singular action of the muscular system; at one time she was bent entirely backwards, resting only on the crown of her head, and her heels as in opisthotonos; again, she was bent forwards, and, finally, she was drawn forcibly to either side. In short, during this attack, all the phenomena of epilepsy, hysteria, and well-marked tetanus, were present."

Soon after this the patient was attacked with variola, and on the last of March she was dismissed in a state of tolerable convalescence. It appears, however, that, on the 19th of April, she returned to the hospital with all her symptoms nearly as violent as ever.

Dr. Duncan appears to have taken considerable pains to prove that, contrary to the doctrine of Cullen, catalepsy may be real, and not feigned. We have not the smallest doubt of the reality of the disease in the foregoing, and in many other cases. We are, at this moment, attending a young lady who has cataleptic attacks every day, and many times during each day. They are generally so transient as to be mostly imperceptible in company—and her great object is to conceal them. They escape notice, except upon particular occasions. Thus, if she is reading, or playing on the piano-forte, the sudden cessation of voice or action is remarked, of course. But if she is merely sitting in company, or joining in general conversation, it is ten to one if the cataleptic suspension of volition be perceived. Once in six or seven days she is seized with a convulsion, in all respects answering to pure epilepsy. The attack commences with a shriek—she falls down—struggles violently—becomes hideously distorted—bites her tongue—and requires two or three attendants to constrain her contortions. She then falls into a profound sleep, and awakes sore and rather poorly, but unconscious, except by these sensations, of what has passed. The complaint has been gradually increasing in violence and frequency, from the age of six to sixteen years. The catamenia have only appeared once, and have not since recurred. Moving in a high sphere of life, this young lady has had the very best—and perhaps the very worst, advice which England could afford. Not the slightest impression has ever been made on the distressing malady—on the contrary, it has progressively augmented in force. Although the intellectual faculties have suffered less than might have been expected, they have not escaped uninjured. The memory is impaired, and application to some particular studies is greatly abridged. She dares not indulge in music—and she is incapable of making the slightest progress in arithmetic. She cannot perform even the most common operations in figures. She delights in history; but the impressions are like those made in water, or, at most in sand. They are soon obliterated. The eyes are expressive—the pupils very large—the complexion exquisitely fair—the features beautiful—the temper mild—and the anxiety to be relieved from her malady intense. The intestinal secretions and excretions are exceedingly depraved—and the catamenia are stopped. These last are phenomena which she cannot feign, if she would;—but who could be so sceptical, or rather so insane, as to imagine that the other distressing phenomena, which deprive her of the pleasures which her rank in life entitle her to, and which she longs for, can be the work of deception? It is preposterous. Her great object is to veil her attacks, and the cataleptic paroxysms usually escape the observation, except of her parents or intimate relations. If she is reading, for example, she will suddenly stop, for an instant—perhaps for half a minute or a minute; being, for that period, like a marble statue—and then she utters a kind of sigh, and takes up the word, or part of the word, where she had stopped. Considering the length of time which the complaint has obtained, we cannot, of course, form any sanguine hopes of recovery. The first object which we have in view, is to correct the alvine derangements and to reproduce the uterine functions. The result of the case we shall freely and candidly communicate to our readers.

To revert to Dr. Duncan's patient. After concluding that her malady was not feigned, and, indeed, could not be feigned, he remarked as follows:—

"The symptoms of the case viewed together, might be arranged under the following heads: 1st, total loss of external perception and sensation; 2dly, suspended volition; 3dly, the continued action of the mixed and involuntary muscles. It was a matter of speculation, whether the functions of the mind were also suspended or not; of their continuance, as yet there had been scarcely any evidence; at the same time there were no grounds sufficiently conclusive of their temporary absence. During the fit, there was a complete semblance, to the view, of sound and healthy sleep; her sleep, however, differed considerably from natural repose; healthy sleep was that, consecutive of exhaustion, or the necessity for which was occasioned by extreme mental exertion; under ordinary circumstances, too, it was gradual in its approach. Morbid sleep, on the other hand, (under which cataleptic and epileptic sleep and somniation were included) was sudden in its seizure, and independent of previous exhaustion or periodical habit; the varieties of morbid sleep were again distinguishable from each other by the mode of resuscitation, the state of the muscular system, the presence or absence of mental phenomena, &c. Thus, in epilepsy, the fit departed gradually, and left the patient in a state of lethargy, or sopor; in catalepsy, the awaking was comparatively immediate, and during the fit the muscles were in the state of rigidity characteristic of the disease; in somniation, the awaking was also sudden, but the sleep was accompanied by speaking, singing, extravagant gesticulations, and other marks denoting the presence and activity of the mental functions. In this patient, therefore, the cataleptic state was indicated by the mode of awaking, the muscular rigidity, the insensibility during the paroxysm, and pain and external noise."

In reviewing the case, on the patient's discharge from the hospital, Dr. Duncan observed that the symptoms and character of the malady had considerably varied during the progress of the complaint. These variations he was inclined to refer rather to a "modified form of hysteria than to any other disease." As the case proceeded, the paroxysms became more and more accompanied by gesticulations, singing, praying, &c. instead of the rigidity of catalepsy. The form of hysteria approached the epileptic, as, in the paroxysms, the patient was totally devoid of consciousness or feeling. The remedies which gave most relief in this case were those which are most useful in hysteria—antispasmodics, narcotics, and purgatives. The shower-bath had been employed two days after her admission, and apparently with some benefit at first; but subsequently with mischief. Powerful purgatives brought away pitchy stools, and temporary relief followed. Indeed, the purgation appeared more beneficial than any of the other remedial measures, till the new symptom of excruciating pain in the abdomen set in, when leeches were applied. This pain was so torturing, that the accession of the cataleptic paroxysm was desirable, as a temporary insensibility to sufferings. The cause of these sufferings Dr. D. was unable to explain—who, indeed, can hope to explain the mysteries of the nervous system in hysteria? One thing is certain, that depletion did not relieve this excruciating pain. Dr. D. alluded to those unaccountable tumours which sometimes shew themselves in hysterical females about the groins, causing great dread of hernia in the minds of medical attendants. He was convinced at any rate that these tumours were *internal*, or situated beneath the muscles constituting the abdominal parietes. In this case flatulence was very distressing. In one instance, after a fright, the spasms became actually tetanic, and lasted four hours, during which time, though the muscular system was in a state of cataleptic rigidity, she sang hymns and repeated the Lord's prayer in a perfectly rational manner. During the eruptive fever of the small-pox, the paroxysms went on; *but were suspended during the presence of the eruption itself*. This was a fortunate circumstance, as a continuance of struggles, during the eruption of pustules on the surface, would have been most distressing. The alcoholic extract of nux vomica was afterwards given in large doses, so as to induce a considerable degree of narcotism. From this time she gradually improved till she was discharged—but whether this improvement resulted from medication, or one of the freaks of the malady, it would be difficult to say. One thing is certain—that, in less than three weeks, she returned in statu quo, and remains in the infirmary. We shall report on both the cases mentioned in this paper on a future occasion.

---

XXXVII.

